# *BraLTP1*, a Lipid Transfer Protein Gene Involved in Epicuticular Wax Deposition, Cell Proliferation and Flower Development in *Brassica napus*

**DOI:** 10.1371/journal.pone.0110272

**Published:** 2014-10-14

**Authors:** Fang Liu, Xiaojuan Xiong, Lei Wu, Donghui Fu, Alice Hayward, Xinhua Zeng, Yinglong Cao, Yuhua Wu, Yunjing Li, Gang Wu

**Affiliations:** 1 Key Laboratory of Oil Crop Biology of the Ministry of Agriculture, Oil Crops Research Institute, Chinese Academy of Agricultural Sciences, Wuhan, China; 2 The Key Laboratory of Crop Physiology, Ecology and Genetic Breeding, Ministry of Education, Agronomy College, Jiangxi Agricultural University, Nanchang, China; 3 Queensland Alliance for Agriculture and Food Innovation, The University of Queensland, Queensland, Australia; National Key Laboratory of Crop Genetic Improvement, China

## Abstract

Plant non-specific lipid transfer proteins (nsLTPs) constitute large multigene families that possess complex physiological functions, many of which remain unclear. This study isolated and characterized the function of a lipid transfer protein gene, *BraLTP1* from *Brassica rapa*, in the important oilseed crops *Brassica napus*. *BraLTP1* encodes a predicted secretory protein, in the little known VI Class of *nsLTP* families. Overexpression of *BnaLTP1* in *B. napus* caused abnormal green coloration and reduced wax deposition on leaves and detailed wax analysis revealed 17–80% reduction in various major wax components, which resulted in significant water-loss relative to wild type. *BnaLTP1* overexpressing leaves exhibited morphological disfiguration and abaxially curled leaf edges, and leaf cross-sections revealed cell overproliferation that was correlated to increased cytokinin levels (tZ, tZR, iP, and iPR) in leaves and high expression of the cytokinin biosynthsis gene *IPT3*. *BnaLTP1*-overexpressing plants also displayed morphological disfiguration of flowers, with early-onset and elongated carpel development and outwardly curled stamen. This was consistent with altered expression of a a number of ABC model genes related to flower development. Together, these results suggest that *BraLTP1* is a new *nsLTP* gene involved in wax production or deposition, with additional direct or indirect effects on cell division and flower development.

## Introduction

Plant non-specific lipid-transfer proteins (nsLTPs) are small, abundant, basic, secreted proteins in higher plants [1], [2]. nsLTPs contain an 8 cysteine motif (8 CM) structure comprising eight cysteine residues linked with four disulphide bonds that stabilize a hydrophobic cavity that allows for the *in vitro* loading of a broad variety of lipid compounds [3], [4].

nsLTPs are encoded by multigene families that were originally subdivided into type I (9 kDa) and type II (7 kDa) on the basis of molecular mass. More recently, several anther specific proteins in Maize (*Zea mays*) and rice (*Oryza sativa*) that displayed considerable homology with nsLTPs that have been proposed as a third type (type III) [5], [6], which differ in the number of amino acid residues interleaved in the 8 CM structure. Recently, fifty two rice *nsLTP* genes, 49 Arabidopsis *nsLTP* genes and 156 putative wheat *nsLTP* genes were identified through genome-wide analyses [7]. Phylogenetic analysis revealed that the rice and *Arabidopsis nsLTPs* cluster into nine different clades, distinguished by a variable number of inter-cysteine amino acid residues [8]. Most studies to date have concentrated on type I, II and III family members, with limited functional analysis of these other six structural types of nsLTPs.

Characterised *nsLTPs* have been implicated in variable and complex physiological functions, mainly related to stress resistance and development, including cuticular wax synthesis [8], [9], [10], [11], abiotic stress [12], [13], [14], disease resistance [9], [15], [16], [17],[18], male reproductive development [19], [20], [21], [22], [23], [24], and cell development [25], [26], [27].

One *nsLTP* family member, a glycosylphosphatidylinositol-anchored lipid transfer protein LTPG, was reported to function either directly or indirectly in cuticular lipid deposition, and mutant plant lines with decreased *LTPG* expression had reduced wax load on the stem surface [10]. Lee *et al* [9] reported that disruption of *LTPG1* gene altered cuticular lipid composition, but not total wax and cutin monomer loads, and caused increased susceptibility to the fungus *Alternaria brassicicola*. Another gene, *LTPG2*, functionally overlaps with *LTPG/LTPG1* during cuticular wax export or accumulation, and the total cuticular wax load was reduced in both *ltpg2* and *ltpg1 ltpg2* siliques [11]. These *LTPG* genes belong to type G *nsLTPs* classified by Edstam *et al* [28], and are not included in Boutrot's classification system [7].

Plant epidermal wax forms a hydrophobic layer covering aerial plant organs. This constitutes a barrier against nonstomatal water loss, as well as biotic stresses, and provides protection against pathogens [29]. Cuticular wax contains very-long-chain fatty acids (VLCFA) and their derivatives, such as alkanes and alcohols, with chain lengths of 20–34 carbons, and wax composition varies with species, organ, and developmental state [30]. Synthesis of VLCFA in the epidermis via acyl-CoA dehydratase PAS2 is essential for the proper control of cell proliferation in *Arabidopsis* [31]. Moreover, it was suggested that VLCFA, or its downstream derivatives or metabolites might function as signaling molecules to suppress cytokinin biosynthesis in the vasculature, thus fine-tuning cell division in internal tissue [31].

Despite progress in the functional analyses of these *nsLTPs* involved in cuticular wax deposition, the exact functions of most *nsLTPs* remain unclear, and complex expression profiles suggest disparate and unpredictable gene functions of unknown *nsLTPs* [22], [23], [27]. Most studies to date have focused on functions of type I, II and III *nsLTP* family members in many species, with few studies on type VI nsLTPs. Thus, the exploration of their roles may prove interesting, especially in non-model or crop species. In this study, we characterized a type VI *nsLTP* gene *BraLTP1* from *Brassica rapa*, and investigated its biological function in the important allotetraploid oil crop, *B. napus*. Our results suggest that *BraLTP1* has the basic characteristics of the *nsLTPs* gene family and is involved in wax deposition, cell proliferation and flower development. To our knowledge few reports linked *nsLTPs* to cell proliferation in plants, and *nsLTP* genes (excluding *LTPG*) involved in wax metabolisms *in vivo* have less been reported. This study will help to deepen our understanding of *nsLTP* family gene function and pave the way for the application of *nsLTP* gene in Brassica breeding.

## Materials and Methods

### Plant material

The plants used in this study were grown in pots containing mixture of moss peat (PINDSTRUP, Danmark) and field soil with the proportion of 3∶1 in a plant growth room set to 20°C±2°C under a 16/8 h photo-period at a light intensity of 44 umol m^−2^ s^−1^ and 60–90% relative humidity.

### Vector construction

**Figure 1:**
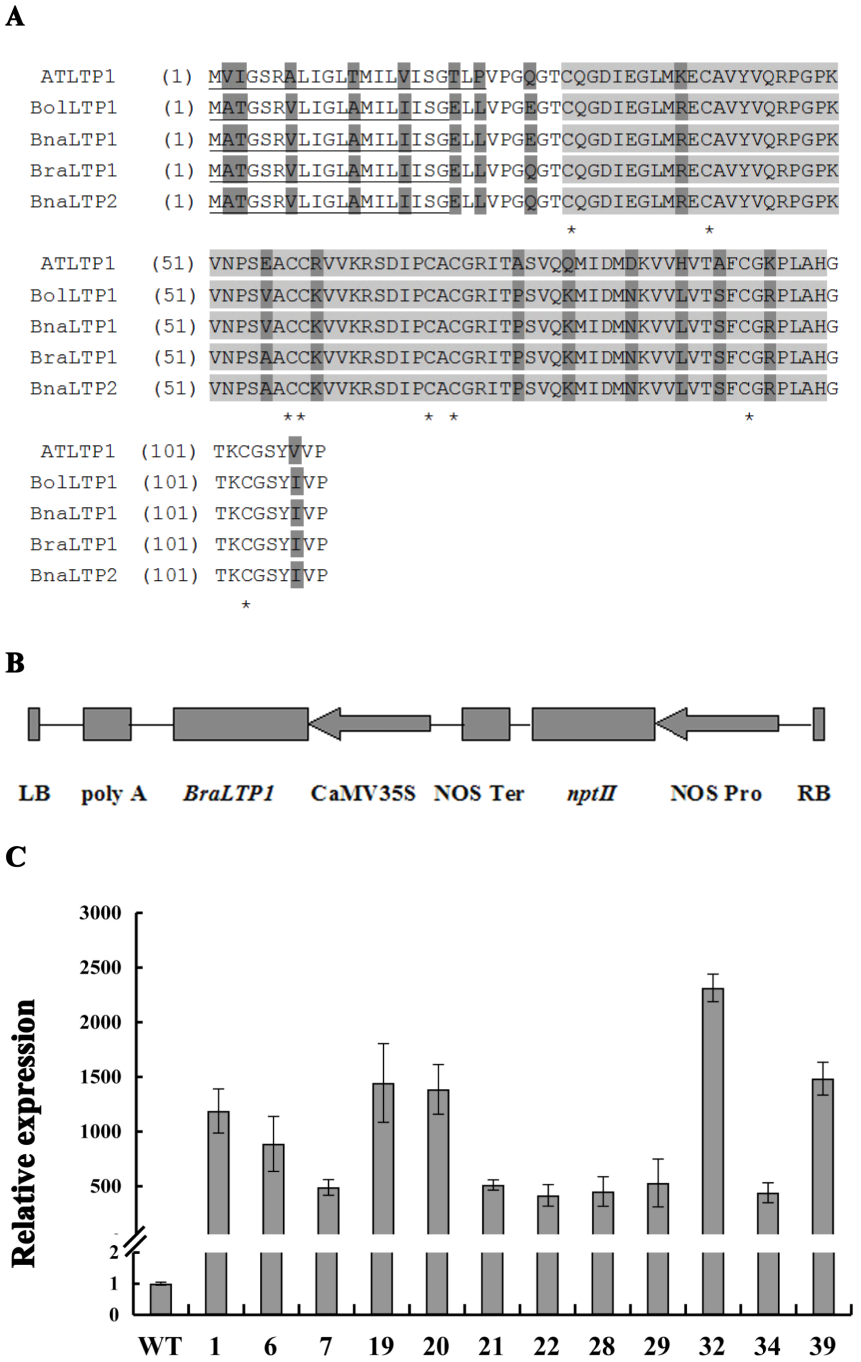
Basic protein characteristics of *BraLTP1* and vector construction. (A) Analysis of the deduced amino acid sequences of BraLTP1 with its homologous sequences in other cruciferae; variable sites (dark grey)the nsLTP-like conserved 8 CM domain (light gray) with conserved cysteine residues (asterisks) and putative extracellular secretory signals (underlined). Sequences are from *Arabidopsis thaliana* AtLTP1 (AT4g30880), *B. rapa BraLTP1* (Bra011229) [32], *B. oleracea* BolLTP1 (Bol018048) [60], *B. napus* BnaLTP1 (AY208878), and *B. napus BnaLTP2* (KM062522). (B) T-DNA region of the *BnaLTP1* overexpression construct containing *BnaLTP1* driven by the CaMV 35S promoter. LB = Left border, RB = Right border, poly A = poly A terminator, *nptII* = kanamycin resistance, NOS Ter = nopaline synthase terminator, NOS Pro = nopaline synthase promoter. (C) Analysis of *BraLTP1* mRNA levels in 10-week-old wild type (WT) and T0 *35S::BraLTP1* transgenic plants by qRTPCR. Transgenic plants include *BraLTP1-1, -6, -7, -19, -20, -21, -22, -28, -29, -32, -34 and -39*. Standard errors were derived from three repeated experiment for the expression levels of each plants.

The coding sequence of *BraLTP1* was amplified from *B. rapa* accession Chiifu genomic DNA using primers designed to the published *B. rapa* sequence Bra011229 (http://brassicadb.org/brad/index.php) [32]. Primers were as follows: BraLTP1-F: 5′- GAGCTCACAACTTCCTTCAAAGCCACA-3′ and BraLTP1-R: 5′-GGATCCCAAACCTCATGGCACAATGTA-3′, containing 5′ restriction enzyme sites for *SacI* and *BamHI* respectively. PCR was carried out in 50 µL, with 50 ng DNA, 0.4 mM dNTPs, 0.2 µM each primer, 0.5 U LA Taq (TaKaRa, Japan) and 1×LA Taq buffer II (TaKaRa, Japan). Conditions were: 94°C for 3 min, 30 cycles at 94°C for 1 min, 55°C for 1 min and 72°C for 1 min. PCR product was checked by gel electrophoresis and target fragment was recovery and purified. The purified PCR product was cleaved using *SacI* and *BamHI*, and ligated between the CaMV 35S promoter and a terminal poly A sequence in the vector PBI121s (Fig. 1B), derived by modifying the multiple cloning site and deleting the GUS gene of PBI121 [33]. Positive clones by PCR using the above gene specific primers were chosen and sequenced (sangon company of Shanghai, China) to make sure they were correct. Standard molecular techniques [34], [35] were used for DNA manipulation.

### Genetic transformation

The *35S::BraLTP1* fragment in PBI121s was introduced into *Agrobacterium tumefaciens* GV3101 by electroporation, and positive clones were selected on on LB agar plates at 37°C, supplemented with appropriate concentration of antibiotics (gentamicin 50 mg L^−1^, rifampicin 50 mg L^−1^ and kanamycin 50 mg L^−1^) and PCR verified. A single positive colony was used to transform *B. napus* cv. Zhongshuang 6, an elite Chinese cultivar in China, as follows: Seeds of Zhongshuang 6 were soaked in 75% ethanol for 1 min and for 10–15 min in a 1.5% mercuric chloride solution. Five to six days after germination under darkness, etiolated hypocotyls were cut in 7 mm segments and mixed with 50 mL *Agrobacterium* in liquid DM media (MS+30 g L^−1^ sucrose+100 µM acetosyringone, pH 5.8) (OD ∼0.3) for 0.5 h. Surface air dried hypocotyls were then transferred to co-cultured medium (MS+30 g L^−1^ sucrose+18 g L^−1^ manitol+1 mg L^−1^ 2, 4-D+0.3 mg L^−1^ kinetin+100 µM acetosyringone+8.5 g agrose, pH 5.8) for 2 days and then to a selection medium (MS+30 g L^−1^ sucrose+18 g L^−1^ manitol+1 mg L^−1^ 2, 4-D+0.3 mg L^−1^ kinetin+20 mg L^−1^ AgNO_3+_8.5 g L^−1^ agrose+25 mg L^−1^ kanamycin+250 mg L^−1^ carbenicillin pH 5.8) for proliferation. After 3 weeks, hypocotyl callus was transferred to regeneration medium (MS+10 g L^−1^ glucose+0.25 g L^−1^ xylose+0.6 g L^−1^ MES hydrate+2 mg L^−1^ zeatin+0.1 mg L^−1^ indole-3-acetic acid_+_8.5 g L^−1^ agrose+25 mg L^−1^ kanamycin+250 mg L^−1^ carbenicillin, pH 5.8) for 2 weeks. Hypocotyls were transferred to new regeneration media every 2 weeks for 3∼4 regeneration cycles before transfer to radication medium (MS+10 g L^−1^ sucrose+10 g L^−1^ agar, pH 5.8) for rooting (about 3 weeks). Transformed plants with roots were transplanted into pots and grown as described. For the construct, more than 60 independent *35S::BraLTP1* T_0_ transgenic plants were generated, and more than 85% were positive transformants as detected using a forward primer designed to the CaMV 35S sequence (35S-F: 5′-AGGACACGCTGAAATCACCA-3) and a reverse primer designed to *BraLTP1* (D-BraLTP1-R: 5′-GGATCCCAAACCTCATGGCACAATGTA-3′). T_1_ seeds of PCR-positive transformants were harvested and grown to T_2_ generation for phenotype identification.

### Protein sequence analysis

*BraLTP1* was aligned to homologous amino acid sequences from several cruciferae including *Arabidopsis*, *B. rapa*, *B. napus* and *B. oleracea*, using Align X multiple sequence alignment software (Vector NTI Advance 11.0, 2008 Invitrogen corporation). Homology search were conducted using BLAST 2.0 program of the National Center of Biotechnology Information (NCBI). Conserved domains were identified using CDD (http://www.ncbi.nlm.nih.gov/cdd/) and the signal peptide was determined by SignalP (http://www.cbs.dtu.dk/services/SignalP/).

### Wax analysis

Epicuticular waxes were extracted from ∼100 mg *B. napus* leaf disks from the fourth fully-expanded leaf from the apex for each plant by mixing in chloroform for 1 min, with 150 µl 100 µg L^−1^ triacontane added as an internal standard. The chloroform was then evaporated under gaseous N2, and the following steps for wax analysis was as described previously [36].

### Water loss determination

For water loss analysis of detached leaves, leaves were detached from 8-week-old plants, placed on a petri dish for 1, 2, 3, 4, 5, and 6 h, and weighed [37]. Three independent experiments were performed, with 5 plants for each line in each experiment.

### Leaf phenotype analysis

*B. napus* leaves were fixed for 24 h in 4% paraformaldehyde. After dehydration using an ethanol series (75% for 4 h, 85% for 2 h, 90% for 2 h, 95% for 1 h and 100% for 30 min twice) the leaves were cleared twice with xylene for 2 h each. The leaves were infiltrated and subsequently embedded in paraffin wax according to the method of Hu *et al* [38]. Seven micromolar sections were obtained using a Leica RM 2016 microtome (Leica, Nanterre Cedex, France) and stained with 1% safranin for 6 h. After dehydration using an ethanol series (50%, 70% and 80% for 3 min each), sections were stained with 0.5% fast green for 30 min and then dehydrated with 100% ethanol for 5 min. Observations were made and images were acquired with a Leica DM 2500 (Leica microsystems, DFC420C). Six leaf disks with 1.5 cm diameter were hole-punched from the 4th leaf of 8-week-old plants and weighed to estimate weight/unit leaf area. For quantification of cytokinin, sampling of ∼100 mg fresh leaves from 4-week-old seedlings was repeated three times, and extraction and determination of cytokinin content were conducted as per Nobusawa et al [31].

### Real-Time PCR

RNA was extracted using a TIANGEN RNAprep Pure Plant Kit (DP 432) according to the manufacturer's instructions. First-strand cDNAs were synthesized from DNaseI-treated total RNA using a TIANGEN FastQuant RT Kit (with gDNase) (KR106) according to the manufacturer's instructions. For transgene expression level and co-segregation experiments, real-time PCR was done using PCR SuperReal PreMix Plus (probe) (TIANGEN). The reaction system and process were followed by the manufacturer's instructions, and four replicates were performed for each cDNA sample. PCR primers and TaqMan probes were designed on the basis of the *BraLTP1* cDNA sequences as follows: sense: RT-BraLTP1-F: 5′-ATCGGTCTAGCAATGATC-3′, RT-BraLTP1-R: anti-sense: 5′-AGCACATTCTCTCATCAG-3′, RT-BraLTP1-probe: 5′-CTCGATGTCTCCTTGGCACG-3′. Specific primers for the *B. napus Actin* gene (GenBank accession number: AF111812.1) were used as an internal control (Actin-F: 5′-CACAGGAAATGCTTCTAAG-3′, Actin-R: 5′-GGATGGATATAGATCGTACC-3′, Actin-probe: 5′-ACTCACCACCACGAACCAGAA-3′). For transcriptional profiling of cell division and flower related genes, real-time PCR was done using PCR SuperReal PreMix Plus (SYBR Green) (TIANGEN). The reaction system and process followed the manufacturer's instructions, as above in four replicates for each cDNA sample. Table 1 provides information about the genes and primers used for the real-time PCR. Specially, *IPT1*, *IPT4*, *IPT6* in *Arabidopsi*s all correspond to the one *Brassica napus IPT* gene. Real-time PCR was performed in an optical 96-well plate with a Bio Rad CFX96 Real-Time System (C1000 Thermal Cycler) (Applied Biosystems, Hercules City, CA, USA).

**Table 1:** Primers for real-time PCR checking genes related to cytokinin synthesis and flower development.

Gene name	Primer name	Sequence(5′→3′)
*IPT1,4,6*	Fna-75600-F	GAGGAGGCAAGTATGGAAGATAG
	Fna-75600-R	CGACGAACTCGAACTCATCATA
*IPT2*	Fna-41524-F	CAAACCAGGAGCTGACTATACC
	Fna-41524-R	AGCGGAGCTATTTGTGTCTG
*IPT3*	Fna-06414-F	TCAGGAATGAGCCGTTCTTAAA
	Fna-06414-R	GTTTGCAAGCTAACCCGAAAG
*IPT5*	Fna-36855-F	GAGCGGAGAAGCGTGATTAT
	Fna-36855-R	CCGACATGCAAGCAAACAG
*IPT7*	Fna-44012-F	TTGGGTCGACGTTTCCTTAC
	Fna-44012-R	GCTTTCGGATCGTGTACTTCT
*IPT8*	Fna-63259-F	GCTTGCCAAGAAGCAGATAGA
	Fna-63259-R	CTCTCTTGACGATGCCCTTAAC
*IPT9*	Fna-63954-F	GCCGTAGACAAAGAGGTGTAAG
	Fna-63954-R	CATTGAGCCAGTGGTACATAGG
*CYP735A1*	Fna-68846-F	GAAACTACCGCACTCCTTCTC
	Fna-68846-R	GCAGCCACATACCTCTCTAATC
*CYP735A2*	Fna-08361-F	CCTCATGCTCCTTGCTCATAA
	Fna-08361-R	TTGCTCAACGGAAGGGATAC
*URH1*	Fna-72547-F	GGGTGGAGACTCAAGGAATATG
	Fna-72547-R	CATGCCACTGATACTGGTGAA
*AP1*	Fna-16396-F	TTCTTAGGGCACAGCAAGAG
	Fna-16396-R	GCATGTATGGATGCTGGATTTG
*AP2*	Fna-27636-F	GCAGATGACGAATTTAACGAAGG
	Fna-27636-R	CTTCCCAACGACCACACTTAT
*AP3*	Fna-72759-F	ATCGAAGGATCACGTGCTTAC
	Fna-72759-R	AATGATGTCAGAGGCAGATGG
*PI*	Fna-60439-F	AAATGTTGGCGGAGGAGAA
	Fna-60439-R	GAATCGGCTGGACTCTGTATC
*AG*	Fna-37522-F	CTGATGCCAGGAGGAACTAAC
	Fna-37522-R	ATGCCGCGACTTGGAAATA
*CRC*	Fna-16399-F	AAGAGTGCCAATCCGGAAATA
	Fna-16399-R	GCTCCGGAAGTAATGGAAGTAG
*SPT*	Fna-54144-F	CTTTGGACCTTTCCCTCACTT
	Fna-54144-R	CATCAAACGCAGCATGTTCTC
*LEUNIG*	Fna-27110-F	ACAGGCAGTGAAGGAGAATG
	Fna-27110-R	CACCCAATTACGAGTAGGGAAG
*AINTEGUMENTA*	Fna-09982-F	AGACACAGATGGACTGGTAGA
	Fna-09982-R	GAGCAGCTTTCTCCTCCATATC
*Actin*	BnActin 88-F	GCTGACCGTATGAGCAAAGA
	BnActin 88-R	AGATGGATCCTCCAATCCAAAC

## Results

### Protein sequence characterization

The *B. rapa LTP1* coding region is predicted to encode a protein of 109 amino acid residues (Fig. 1A). Similarity searches revealed that *BraLTP1* has 84% overall amino acid identity with *AtLTP1* of *Arabidopsis*, 98% identity with both *BolLTP1* from *Brassica oleracea* and *BnaLTP1* from *B. napus* (which are 100% similar to each other), and 100% identity with *BnaLTP2* of *B. Napus* (while gene sequences were 98% similar). *B. napus* (*Brassica* AC genome) is an allotetraploid species resulting from a cross between *B. rapa* (A genome) and *B. oleracea* (C genome) and thus it is not surprising that it contains LTP protein copies corresponding 100% to the A and C genomes derived from both of these diploid species (Nagaharu., 1935). All species possessed highest levels of sequence similarity in the *nsLTP*-like domain regions (light shaded amino acids 29 to 99) (Fig. 1A). A putative extracellular secretary signal (amino acids 1 to 22 in *Arabidopsis* and 1–19 in the other *Brassica* species) was 100% conserved in the *Brassica* species (Fig. 1A). Nine amino acid substitutions exist in the *nsLTP*-like domains between *AtLTP1* and the *Brassica* genes. The eight strictly conserved cysteine residues in all plant LTPs that form four intrachain disulfide bridges, were also 100% conserved among all aligned sequences.

In *Arabidopsis*, nine classes of *nsLTP* were identified that contain a variable number of inter-cysteine amino acid residues [7]. The number of amino acids between the eight cysteines in the 8 CM motif of *BrLTP1* and it's aligned sequences are 10, 16, 0, 9, 1, 22 and 9, with a methionine and a valine residue present 10 and 4 aa before Cys7. These characteristics place the *BraLTP1*, *AtLTP1, BolLTP1*, and *BnaLTP1/2* proteins as secreted LTPs in class VI of plant *nsLTPs*.

### Overexpression of *BraLTP1* in *B. napus*

**Figure 2:**
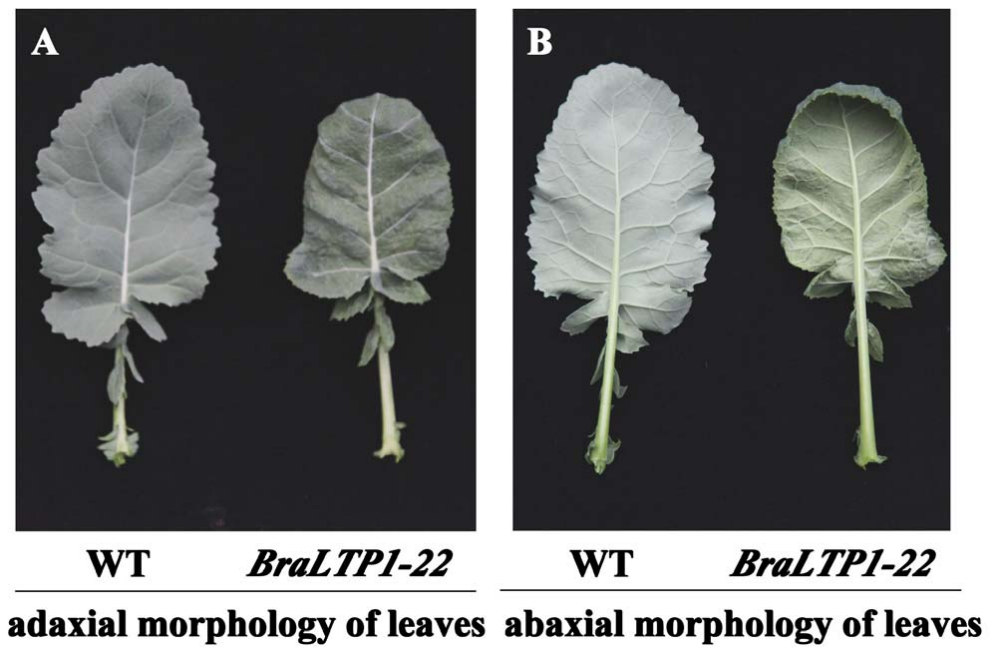
Leaf morphology of *35S::BraLTP1* overexpression line 22 (*BraLTP-22*). The adaxial (A) and abaxial (underside) (B) morphology of a representative wild type (WT) leaf (left) and *35S::BnaLPT1* (right) are shown. WT leaves appear glaucous with a smooth surface compared to *35S::BnaLTP1*, which has an unglaucous, dark green and bumpy leaf surface with abaxial edge-curling.

**Figure 3:**
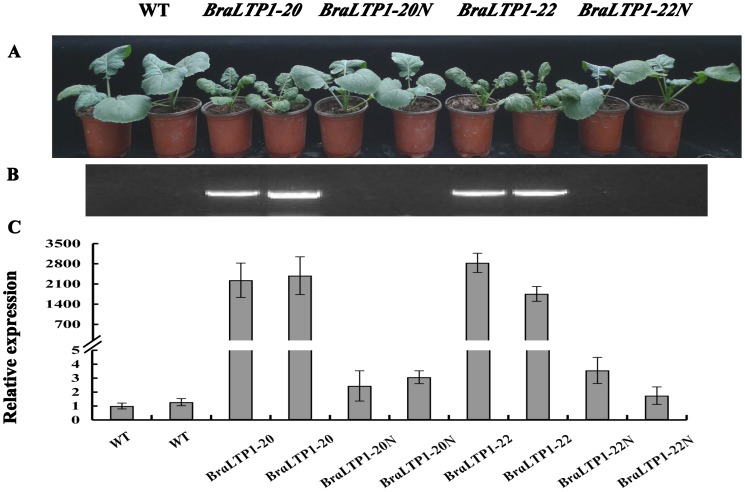
Cosegregation experiments of two *35S::BraLTP1* transgenic lines. (A) Phenotype of 5-week-old T1 plants segregating for *BraLTP1*-20 and *BraLTP1*-22. (B) PCR results using primers spanning the CaMV 35S promoter and *BraLTP1* in the vector, with DNA of corresponding plant in panel (A) as templates. (C) Transcript level of *BraLTP1* for each plant shown directly above in panel (A). PCR-positive transformants show high *BraLTP1*-expression and displayed the typical overexpression phenotypes including dark-green, wrinkled, curly leaves and smaller stature compared with their negative segregants (*BraLTP1-20N* and *BraLTP1-22N*).

Given the 100% similarity between the *B. rapa LTP1* and *B. napus LTP2* proteins, and the relative agronomic importance of *B. napus*, we carried out functional analysis of *BraLTP1* in *B. napus*. Overexpression of *BraLTP1* driven by the CaMV 35S promoter in 12 independent transformants of the *B. napus L.* cultivar Zhongshuang 6 showed expression levels 415 times to 2314 times higher than in wild type using qRTPCR (our primers could analyse *BraLTP1* from *Brassica rapa* and its homologous gene from *Brassica napus* without difference) (Fig. 1C). The most visually striking feature of the *BraLTP1* overexpression lines was a distinctly green leaf phenotype, with no waxy surface visible on either the abaxial or adaxial surfaces compared with the wild type (Fig. 2). Additional morphological differences in *BraLTP1* overexpressing plants included humpy and wrinkled leaf surfaces, abaxially curvature of leaf edges, and a significant reduction in total plant size relative to the wild type and negative segregants (Fig. 2 and Fig. 3A). These phenotypes were widespread in most lines, and we chose two *35S::BraLTP1* transgenic lines (*BraLTP1-20* and *BraLTP1-22*) for further study due to their low copy numbers, morphologically clear phenotype and high transgene expression, enabling easy generation and comparison to negative segregates as controls.

To confirm that these phenotypes resulted from specific over-expression of *BraLTP1* rather than tissue culture or vector insertion effects, we performed co-segregation analysis. Leaf samples of positive and negative segregants of *BraLTP1-20* and *BraLTP1-22* lines, together with wild type, were selected based on genomic PCR for the insert (Fig. 3B) and real-time PCR of *BraLTP1* expression (Fig. 3C). Plants of *BraLTP1*-20 and *BraLTP1*-22 that were positive for the insert and had high *BraLTP1* transcript level also had the phenotypes described above, while negative segregants of these lines showed the same phenotype as the wild type controls (Fig. 3A, B and C). Therefore, *35S::BraLTP1* transgenic phenotypes perfectly cosegregated with overexpression of the *BraLTP1* gene.

### Overexpressing *BraLTP1* reduces cuticular wax in leaves

**Figure 4:**
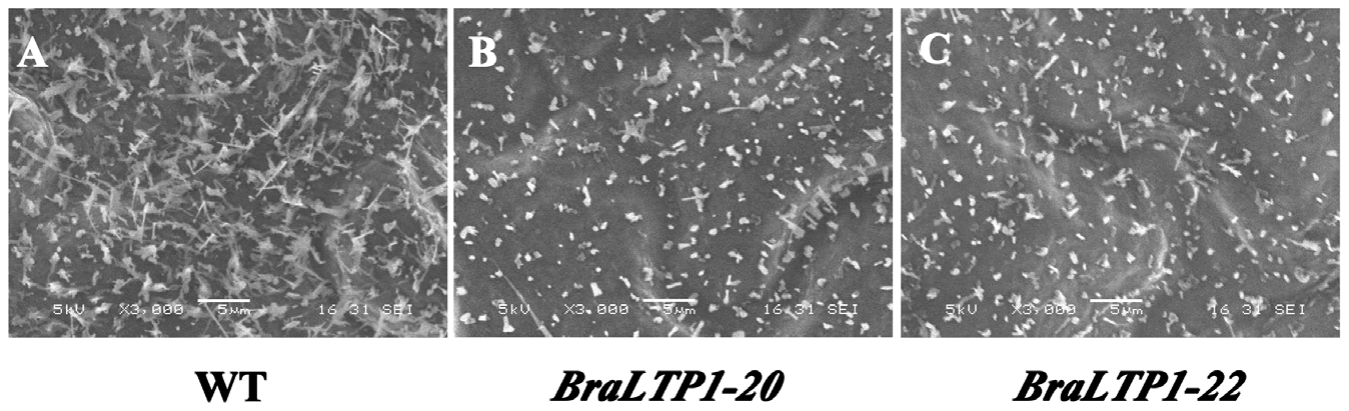
Epicuticular wax and cuticle layer of air-dried adaxial leaf surfaces of wild type (WT), the *BraLTP1-20* and *BraLTP1-22* overexpressor using scanning electron microscopy. The experiments were repeated for 3 times with at least five plants for each time. (A) Wax crystals on the WT leaf were dense, with high proportion of tubular-like wax crystals. (B and C) Wax crystals are sparsely distributed on *BraLTP1-20 and BraLTP1-22 B. napus* leaves. Bar = 5 µm.

Mutant analyses have implicated a role for *nsLTPs* in the transport of waxes or cutin monomers [8], [9], [10], [11], however overexpression of *nsLTPs* has seldom been reported. To determine whether the visible leaf phenotypes of the *B. napus BraLTP1* overexpressor lines resulted from decreased cuticle wax, the density of wax crystals on the leaf surface was assessed by scanning electron microscopy (SEM). A clear reduction in wax crystal density was observed on leaves of the *BraLTP1*-22 plants, accompanied by altered crystal shape and form (Fig. 4). This suggested that aletred epicuticular wax might lead to the visible phenotypes of *BraLTP1* overexpressing leaves.

**Figure 5:**
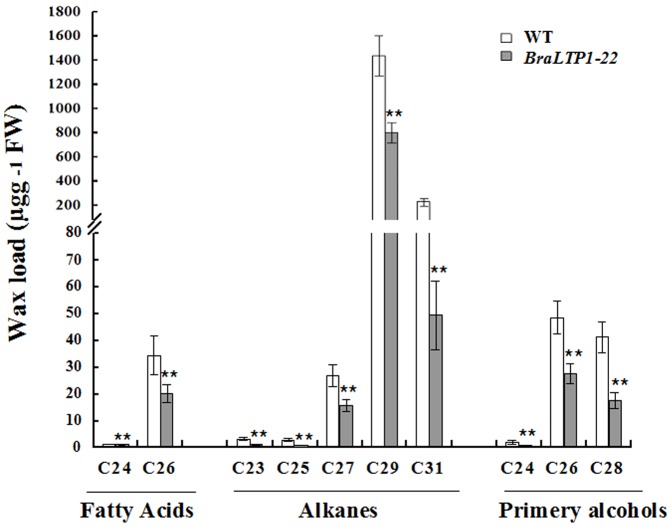
Cuticular wax composition and loads in leaves of *BnaLTP1-22* overexpression line and wild type (WT) *B. napus*. Error bars indicate SE of three 6 biological repeats (t-test: **P<0.01).

To determine the chemical composition of *BraLTP1* overexpressing leaves in greater detail, gas chromatography mass spectrometry (GC-MS) analyses were performed. A 78% reduction in levels of the C31 alkane, hentriacontane, a major component of cuticular waxes, was seen in *BraLTP1*-22 leaves (49.6 µg g^−1^) relative to wild type (221.3 µg g^−1^). A second major wax constitutent, C29 alkane (nonacosane), was decreased by 44% in the overexpressor line relative to wild type; from 1431.7 µg g^−1^ to 82.8 µg g^−1^. Other wax components were similarly reduced, ranging from 17% to 80% reductions (Fig. 5). Despite these defects in cuticular wax, *BraLTP1*-22 overexpressors did not display any organ fusions, unlike some other mutants with cuticle defects [39], [40]. Together these results suggest a broad-range, non-specific reduction in wax deposition in *B. napus*.plants overexpressing *BraLTP1*.

**Figure 6:**
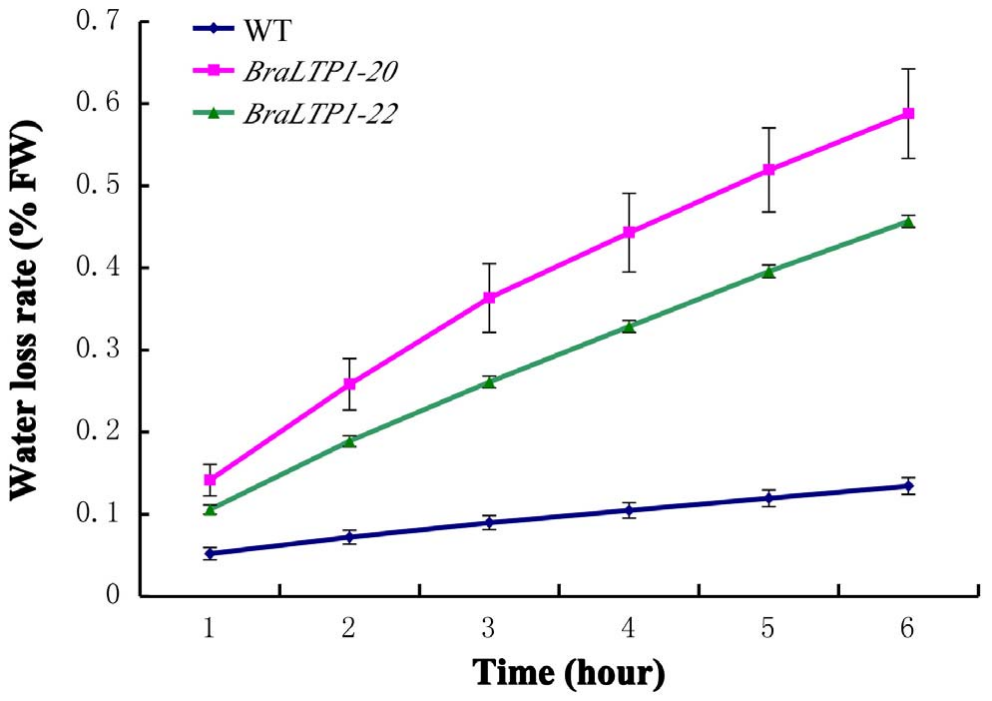
Water loss of detached leaves of wild type (WT), *BraLTP1-20* and *BraLTP1-22* plants. The data are the means ± SD of three replicates (*n* = 5 for each experiment); *P*<0.01 (ANOVA, P = 6.53×10^−56^).

Distortion of the cuticular layer often results in an increased permeability of leaves [41], [42], [43]. To test this, water loss assays of detached leaves from *BraLTP1*-20, *BraLTP1*-22 and wild type were performed. A significantly higher rate of water loss occurred for the detached leaves of *BraLTP* overexpressing lines when compared with wild type (Fig. 6). This is consistent with the observed abnormal cuticular layer of overexpressor leaves, suggesting that wax defects can result in perturbed cuticle permeability. The result also hints that *BraLTP1* could be important for plant water relations and drought tolerance.

### Overexpression of *BraLTP1* promotes cell overproliferation in leaf

**Figure 7:**
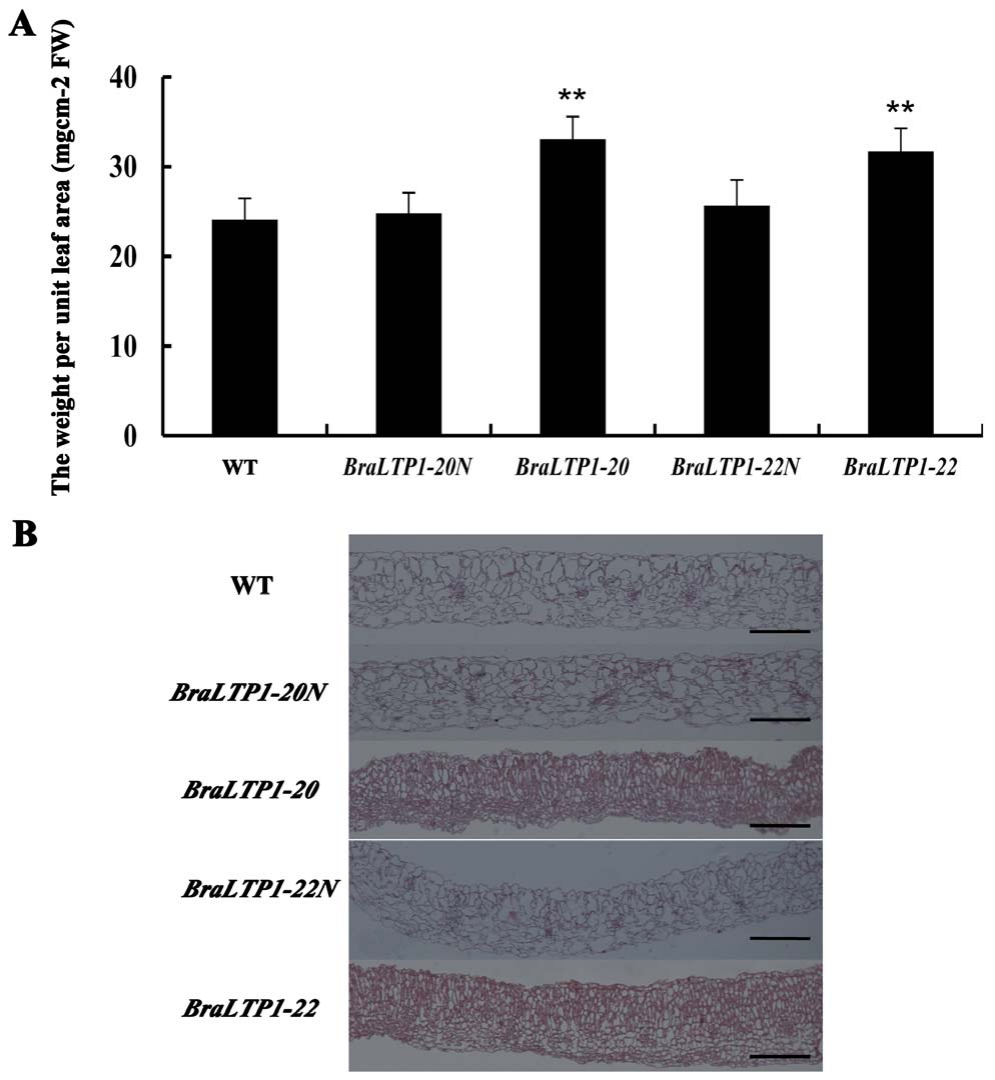
Overexpression of *BraLTP1* promotes cell proliferation. (A) The weight of per unit leaf area of wild type (WT), *BraLTP1*-20 and *BraLTP1*-22 and their segregated negative controls *BraLTP1*-20N and *BraLTP1*-22N. Leaf disks were collected from the fourth fully-expanded leaf from the apex taken from 8-week-old plants. Data are the mean±SD from three independent experiments using leaves of five plants. **Significant differences at the levels of P<0.01. (B) Representative leaf cross-sections of segregating *35S::BraLTP1* and WT plants. The fourth fully-expanded leaf from the apex were taken from 6-week old plants of WT, *35S::BraLTP1* transgenic plants (*BraLTP1*-20 and *BraLTP1*-22), and null segregates of these two lines (*BraLTP1*-20N and *BraLTP1*-22N). The experiments were repeated for 3 times. Bar = 200 µm.

In addition to the wax phenotype, the cellular morphology and weight of per unit leaf area was examined (Fig. 7A and B). *BraLTP1*-20 and *BraLTP1*-22 leaves were 0.033 g cm^−2^ and 0.031 g cm^−2^ respectively, significantly higher than negative segregants and wild type, which varied from 0.024 g cm^−2^ to 0.026 g cm^−2^ (P<0.01) (Fig. 7A). To examine changes at the cellular, structural level, paraffin-wax-embedded leaf cross-sections stained with Safranin and fast green were examined. This demonstrated that in *BraLTP1* overexpressing lines, the cellular layer of both palisade tissue and parenchyma tissue was increased; with palisade cells, parenchyma cells and epidermic cells smaller and more compact than negative segregants and wild type sections (Fig. 7B). Thus, increased cellular layering and a compact cell arrangement likely led to the increase in weight for per unit leaf area. This underlying change in leaf cell layer number and density likely contributes to the visible morphological defects including leaf curling.

**Figure 8:**
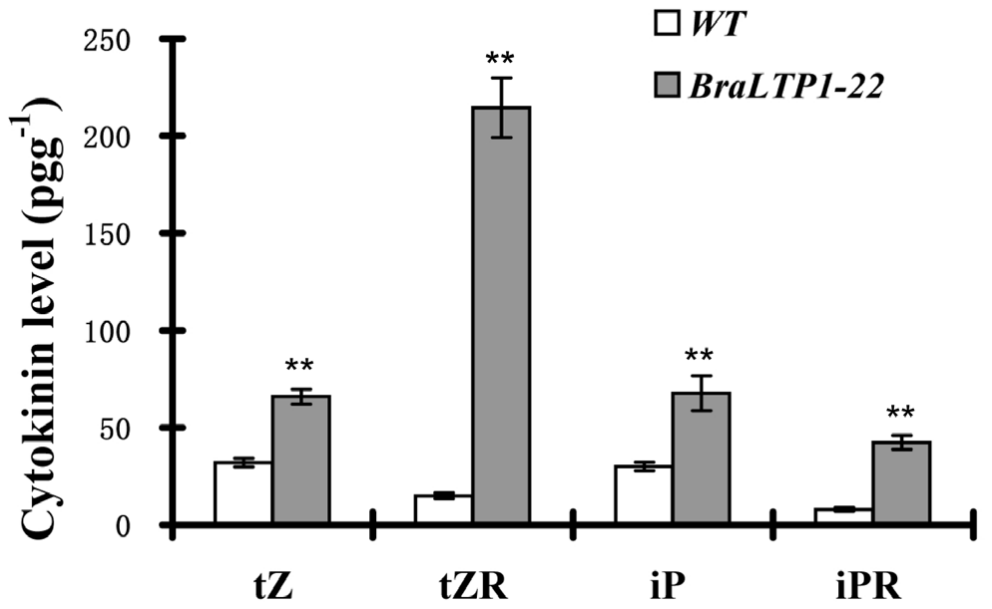
Cytokinin content in *BraLTP1* overexpressing leaves. Amounts of tZ, tZR, iP, and iPR were measured in leaves of 4-week-old *BraLTP1-22* and wild type (WT). Data are presented as mean±SD (n = 3).

To correlate these changes to cytokinin composition, we quantified cytokinin content, including the levels of the cytokinins isopentenyladenine (iP) and trans-zeatin (tZ), and of their riboside (iPR and tZR), As observed in *BraLTP1-22* line, the *BraLTP1* overexpressing leaves contained significantly higher amounts of tZ, tZR, iP, and iPR when compared with the wild type (P<0.01, Fig. 8), consistent with more cell accumulation in leaves (Fig. 7B). This indicates that active cytokinins are highly synthesized in leaves overexpressing *BraLTP1*.

### *BraLTP1* affects flower development

**Figure 9:**
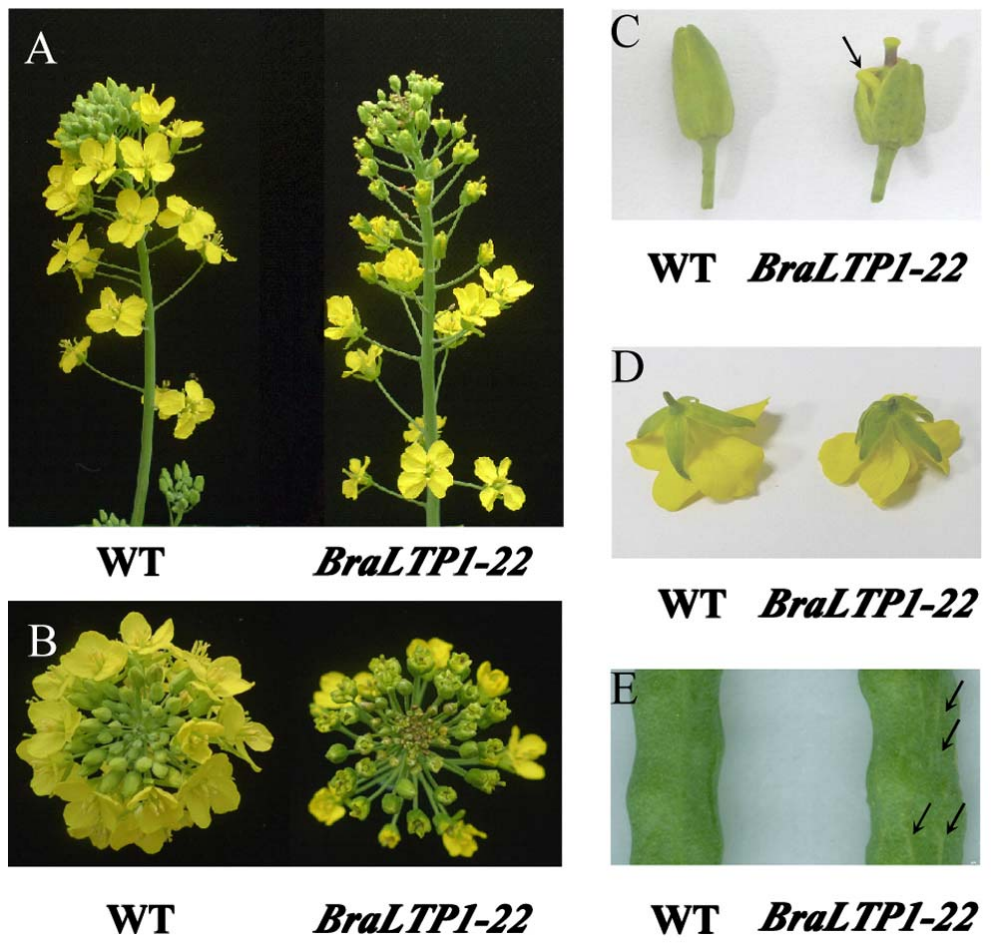
Morphological deformities of *BraLTP1* overexpressing flowers. (A) Lateral view of wild type (WT) and *35S::BraLTP1* (*BraLTP1-22*) primary inflorescences. (B) Top view of WT and *35S::BraLTP1* (*BraLTP1-22*) inflorescences. (C and D) elongated carpel, outward bending stamen (arrow indicated), and disorganized sepals of open (D) and budded (C) flowers of *35S::BraLTP1*(*BraLTP1-22*) vs normal development of WT plants. (E) Silique epidermis of *35S::BraLTP1* (*BraLTP1-22*) and WT plants; arrow indicates the longitudinal ridges that all disturbed in different places.

In the reproductive phase of the *B. napus* lifecycle, overexpressing *BraLTP1* resulted in early development of longer carpels and outward bending stamen in the flower. Early in flower development, carpels grew out of the apical flower buds, with the outward bending stamens clearly visible from the slightly opened sepals, giving an observably distinct phenotype from wild type flowers (Fig. 9A, B and C). Microscopic examination of the petals in the bud showed a variable severity of this phenotype in different lines. In the moderate version of this phenotype, the four petals developed into nearly normal petals upon flower opening (Fig 9A, B and D), while in severe phenotype, poorly developed, shriveled petals were found resulting in only one to three petals in the opened flowers (data not show). These phenotypes occurred not only in primary inflorescences but also in other branched inflorescences. In addition, bumpy sepals and siliques with all distributed longitudinal ridges on the surface, similarly to the bumpy phenotype of leaves, were observed in *35S::BraLTP1* transgenic *B. napus* lines (Fig. 9D and E). Cross-section of sepals and siliques did not show a difference in cell proliferation between transgenic plants and wild type (data not show). Microscopic examination of the ovules within the carpels revealed developing embryos and endosperm, indicating self-compatibility and good fertilization; pollen viability identification revealed that pollen production was also normal (data not show). Silique filling occurred effectively, thus *BraLTP1* over-expression had few detrimental effects on plant fertility (Fig. 9E).

### Co-regulated genes to cytokinin synthesis and flower organ development

The transcript abundance of 10 genes involved in the cytokinin synthesis pathway, and 13 genes involved in floral organ development, were investigated by real-time PCR in the wrinkled leaves of *BraLTP1* overexpression lines as well as in wild type (Table 1).

**Figure 10:**
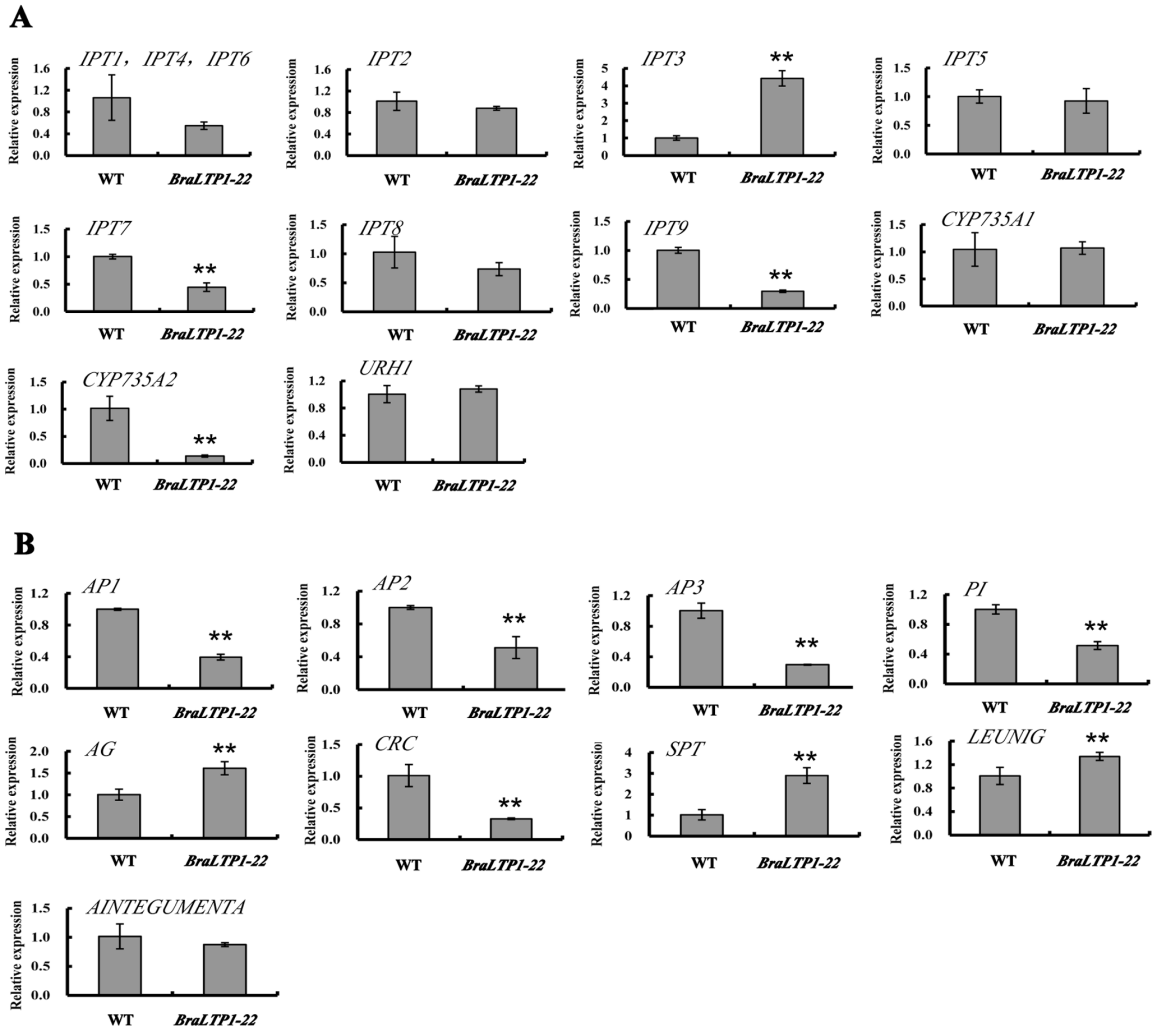
Transcript abundance of various cyctokinin-related (A) and flowering-related (B) genes in *35S::BraLTP1* (*BraLTP1-22*) and wild type (WT) plants as determined by qRTPCR. Data is the average of three plants with standard errors. Asterixes indicate significant differences to WT (** P<0.01).

In *Arabidopsis*, seven genes for adenosine phosphates isopentenyl transferase (*AtIPT1* and *AtIPT3* to *AtIPT8*) have been identified as cytokinin biosynthesis genes [44], [45]. *IPT* and *CYP735A*, are responsible for the differential distribution of *de novo* synthesis pathways for isopentenyladenine (iP), trans zeatin (tZ) [46]. Recently, a novel uridine ribohydrolase, *URH1*, was characterized that degrades isopentenyladenosine in *Arabidopsis* [47]. In leaves of *BraLTP1* overexpression lines, the cytokinin biosynthesis genes *IPT3* were significantly increased (P<0.01), with 3.8 times of expression levels in wild type, while *IPT7*, *IPT9* and *CYP735A2* were decreased (P<0.01), and there was no significant change in *IPT1, IPT2, IPT4, IPT5, IPT6, IPT8, CYP735A1* and *URH1* (P>0.05) (Fig. 10A).

The ABC model of flower development describes how the combinatorial interaction of three classes of genes directs the development of four types of floral organs [48], [49]. Here, in *BraLTP1* overexpression lines, the expression level of genes involved in flower development including *AP1*, *AP2*, *AP3*, *PI*, *CRC* were significantly decreased in the abnormally-developed flowers, while *AG*, *SPT* and *LEUNIG* were significantly increased. There was no significant change in *AINTEGUMENTA* (Fig. 10B).

The decreased transcript levels of *AP1, AP2, AP3, PI* are consistent with the phenotype of *BraLTP1* overexpressing flowers in *B. napus*, with short and humpy sepals and outwardly bent stamens. Furthermore, the increased expression levels of *AG*, *SPT* and *LEUNIG* were consistent with the over-developed longer carpels. These results, plus phenotypic data suggest that *BraLTP1* plays a role in flower development.

## Discussion

### Functional characterization of *BraLTP1*

In this study, we report the isolation and characterization of an *nsLTP*-like gene from *B. rapa*; *BraLTP1*. Sequence analysis showed that the *BraLTP1* protein is a single copy gene in the ‘A’ genome of *B. rapa*, with a homologous gene in *B. olercaea*, ‘C’ genome and two corresponding ‘A’ and ‘C’ genome copies in the amphidiploid ‘AC’ genome of *B. napus*. *BnaLTP1* was first reported by Dong [50] as a *B. napus* seed specific gene named *Bn15D18B* (genbank number: AY208878), which was differentially screened in a seed-cDNA library harvested 15 days after pollination (DAP). The amino acid similarity of *Arabidopsis*, *B. rapa*, *B. oleracea* and *B. napus LTP1* copies is high and extends throughout the whole protein, with increasing divergence consistent with the older evolutionary relationship of Arabidopsis. The configuration of the 8CM domain and inter-cysteine amino acid residues places *BraLTP1* in class VI of *nsLTP*, which in A*rabidopsis* is composed predominantly of uncharacterized proteins including *At1g32280.1*, *At4g30880.1*, *At4g33550*, and *At5g56480.1* [7]. Until recently, all type VI *nsLTP* genes were less studied, with unknown functions, providing good opportunity to expose new physiological functions of this family in processes such as cell division, as shown herein.

In this study, we cloned and functionally analysed a type VI *nsLTP* in *B. napus*. Over-expression of the *BraLTP1* gene caused growth defects in the seedling and reproductive organs of *B. napus*. These included a distinct green, disorganized leaf surface with curled edges and abnormally developed flower. Decreased levels of epicuticular wax accumulated on the leaf epidermis, and cell layering and cell density were increased in the mesophyll cell of *BraLTP1* overexpressing leaves. Increased cytokinin levels including of tZ, tZR, iP, and iPR and increased expression of the cytokinin-synthesis gene *IPT3* in *BraLTP1* overexpression lines correlated well with the enhanced cell proliferation phenotype. Overexpression of *BraLTP1* also led to the altered transcription of many important ABC model flower development genes, coinciding with the visible morphological and developmental perturbations in these lines. Overall, our experiments suggest that *BraLTP1* is an important *nsLTP* gene affecting wax deposition, cell proliferation, and leaf and flower morphology development in *B. napus*.

### Overexpressing *BraLTP1* leads to a reduction of wax load

*NsLTPs* are proposed to play a role in the delivery of wax components during the assembly of the cuticle [26], [51]. Previous studies suggested that in *nsLTP* mutants such as *ltpg1*, *ltpg2* and *ltpg1ltpg2*, wax load decreases with the reduced expression of *nsLTP* genes [10], [11], [12]. Therefore, we hypothesised that overexpressing *nsLTP1* in *B. napus* might lead to wax enrichment. However, in *BraLTP1* overexpressing plants, wax accumulation was decreased in seedling leaves. The amount of wax on *BraLTP1-22* leaves was significantly reduced with proportional deficiencies in the component aldehydes, alkanes, alcohols and acids compared with wild type. This universal reduction, with no specific component alteration, in *35S::BraLTP1* transgenic plants suggested that the effect of *BraLTP1* overactivity was not substrate specific.

There are a few possible explanations for this contradicting observation: (1) the genes above belong to glycosylphosphatidylinositol-anchored *LTPs*, which are different from the type VI *BraLTP1* and exercise different mechanisms *in vivo*; (2) overexpression of *BraLTP1* may lead to disordered or destructive secretion of wax out of cells, which is subsequently lost from the surface; (3) overexpression of *BraLTP1* gene somehow feedback inhibits, or competitively inhibits, other *nsLTPs* with important complimentary lipid synthesis or transport abilities. Until recently, functional analysis of wax-synthesis-related *nsLTP* genes has focused on mutants, while transgenic overexpression is seldom reported. To our knowledge the only prior overexpression study was on *LTP3* in *Arabidopsis*, overexpression of *LTP3* enhanced freezing and drought tolerance in *Arabidopsis* but with no change of cuticular wax seen [15]. Further investigation of the molecular mechanism of *BraLTP1* action will shed more light on its function in wax metabolism and feedback regulation.

### Overexpressing *BraLTP1* leads to cell overproliferation

Besides wax load reduction, we also found that (1) *35S::BraLTP1* transgenic *B. napus* plants exhibit disorganized leaf patterning/morphology; (2) mesophyll cells were over proliferated, with increased cell layer number and cell density, and; (3) cytokinin levels were significantly increased and (4) the cytokinin-synthesis gene *IPT3* was increased 3.8 fold in transgenic leaves. It is known that wax is composed of VLCFA and their derivatives, thus our data is consistent with Nobusawa *et al* [31], wherein VLCFA synthesis in the epidermis confines cytokinin biosynthesis via *IPT3* to the vasculature and restricts cell proliferation. While it is also possible that *BraLTP1* itself plays a direct role in cell division by affecting related genes. However, the internal mechanism remains to be clarified.

The decreased expression of other cytokinin synthesis related genes seen here may be due to feedback downregulation through the pathway, or functionally differentiated roles of *IPTs* in response to environmental conditions. VLCVA, its derivatives, or VLCFA-related lipids, may function as signaling molecules to control cell division by affecting cytokinin related gene transcription [31], [52], [53], [54]. Further studies are needed to examine such possibilities and explore the specific mediators or ligands which suppress cell proliferation in tissues. Mutant material in Nobusawa's study was difficult to observe macroscopically because of severe growth defects and in our experiment, overexpressing line for *BraLTP1* gene analysis produced a moderate, morphologically observable phenotype that produced fertile seed for continued research, shedding important light on the system for study of plant-enviroment interaction.

### Overexpressing *BraLTP1* led to altered flower morphology

Some studies have reported that *nsLTPs* are involved in flower development. For example, multiple *nsLTP* genes were identified to be differentially expressed in petals during different developmental periods in carnation flowers, suggesting their contributions to petal development [55]. *FIL1*, a non-specific lipid-transfer protein with an *nsLTP*-like domain was reported to be important in petal and stamen formation in *Antirrhinum* [56]. The identification of *Antirrhinum nsLTPs* as target genes of the class B MADS box transcription factors *DEFICIENS*, suggested a function during late petal and stamen development [57]. Kotilainen *et al* [58] reported that the *gltpl* gene in *Gerbera hybrida* var. Regina was expressed only in the corolla and carpels and was developmentally regulated during corolla development.

In the ABC model of flower development, the A-class genes *APETALA1* (*AP1*) and *AP2* confer sepal. Their activity overlaps with B-class genes *APETALA3* (*AP3*) and *PISTILLATA* (*PI*), which develops into petals. B-class genes and the C-class gene *AGAMOUS* (*AG*) specify stamen, while *AG* promotes carpel development [48], [49]. Two newly characterized genes, *CRABS CLAW* (*CRC*) and *SPATULA* (*SPT*), function similarly to AG to promote carpel differentiation. *LEUNIG* and *AINTEGUMENTA* are also putative genes affecting carpel development [59]. In our study, overexpressing *BraLTP1* led to altered expression levels of class ABC genes, which control flower organogenesis, with *AP1*, *AP2*, *AP3*, *PI* decreased. This is consistent with the morphological defects of sepals and stamens seen. The expression level of *AG*, *SPT* and *LEUNIG* were increased, which may result in the early development of longer carpels (Fig. 10B). Combined with previous studies of *nsLTP* on flower development, we speculate that *BraLTP1* may affect flower development through the regulation of morphologically important cellular components like the cell wall and cuticle, to affect flower-related genes.

### Conclusion

This study identifies a novel *nsLTP* gene *BraLTP1* that influences wax deposition, cell proliferation and flower development when overexpressed in *B. napus*. Although the precise biological role is yet to be determined, we suggest that *BraLTP1* may link the metabolism of wax lipids and/or cell wall components in the epicuticular, or internal plant interfaces, to the coordinated execution of developmental programs, including cell division and flower development. Therefore, *BraLTP1* likely plays important roles in different developmental periods in plants.
